# Exploring the Impacts of Preventative Health Behaviors with Respect to COVID-19: An Altruistic Perspective

**DOI:** 10.3390/ijerph19137573

**Published:** 2022-06-21

**Authors:** Yi-Fang Luo, Shu-Ching Yang, Shih-Chieh Hung, Kun-Yi Chou

**Affiliations:** 1 Intelligent Electronic Commerce Research Center, Institute of Education, National Sun Yat-Sen University, Kaohsiung 80424, Taiwan; a0989909301@gmail.com (Y.-F.L.); chouky0914@gmail.com (K.-Y.C.); 2 Center for Teaching and Learning Development, National Kaohsiung University of Science and Technology, Kaohsiung 805301, Taiwan; 3 Department of Information and Communication, Southern Taiwan University of Science and Technology, Tainan 710301, Taiwan

**Keywords:** altruism, COVID-19 preventive health behaviors, well-being, depression

## Abstract

This study aims to explore the impact of gender and anxiety on various preventative health behaviors, and the relationships among these preventative health behaviors, individual well-being and depression, from the perspective of altruism. This study employed an online questionnaire survey, and 136 males and 204 females participated in the survey. The results of this study showed that females exhibited better preventative health behaviors than males, including hygiene habits, social distancing and behaviors intended to help others mitigate the epidemic. Anxiety regarding COVID-19 infection encouraged individuals to adopt hygienic habits and social distancing measures rather than to help others mitigate the epidemic. Hygiene habits improved the individual’s psychological well-being. Helping others mitigate the epidemic improved the individual’s psychological well-being and social well-being and contributed to reducing individual depression. However, the preventative health behavior involved in social distancing was not conducive to emotional well-being or social well-being. Affective elements are related to individual behaviors. Therefore, the use of prosocial, altruistic language may play an important role with respect to encouraging people to comply with preventative health behaviors in the context of COVID-19. In addition, it is worth noting that different preventative health behaviors may have different effects on people’s mental health, especially when implementing social distancing-related epidemic mitigation behaviors. The question of how to prevent negative psychological effects in restricted actors must be answered, and the degree of life satisfaction experienced by those actors must also be taken into account.

## 1. Introduction

COVID-19 is regarded as the most devastating infectious disease of this century, not only due to widespread contagion, but also because of the severe complications it causes [1]. Factors which caused the COVID-19 outbreak to be a major international public health emergency also gave rise to a great deal of public concern [2]. Relying on government policy measures alone is insufficient for complete control of the spread of the epidemic. To reduce the spread of infectious diseases and understand the public’s response to COVID-19, effective risk communication and epidemic control and prevention strategies are crucial [3]. In particular, the adoption of preventative health behaviors was seen as key to combating the spread of COVID-19 in the absence of a vaccine [4,5,6]. In the early stage of the global spread of the epidemic, Taiwan adopted a strict predefense strategy, including restricting the purchase of masks to consolidate supplies, mandatory wearing of masks for public transportation passengers, school postponement, and requiring home quarantine for inbound passengers [7].

From a public policy perspective, preventative health behaviors (e.g., following social distancing guidelines, washing hands, and wearing masks) provide both private and public benefits by reducing the risk of individual infection while also creating opportunities for positive externalities that help reduce the risk of infection for others. Therefore, preventative health behaviors are also regarded as manifestations of altruism [8,9]. Campos-Mercade, Meier, Schneider, and Wengström also demonstrated that altruists are more likely to comply with social distancing and hygiene recommendations as well as to donate to charity during the COVID-19 outbreak [10]. Wang et al. found that the main reason for adolescents to comply with social distancing norms is to protect others rather than to protect themselves, which is a manifestation of altruistic behavior [11]. Altruistic behavior is voluntary behavior that benefits others [12]. However, altruism is not always opposed to self-interest. Reciprocal altruism holds that as long as reciprocity is involved, people who pursue self-interest may also engage in behaviors that benefit others [13,14]. Prosocial behavior [15], a term that is often used interchangeably with altruism, includes both one-way acts that help others and instances of bidirectional social interaction. That is, individuals are aware of the benefits of being with others, so they engage in corresponding cooperative behaviors. Although such behaviors may require the individual to pay and sacrifice, the individual is still willing to cooperate to benefit others [16].

From the perspective of utility maximization, utilitarianism pursues the principle of greatest happiness [17]. In the medical and health field, “happiness” is usually replaced by the concept of “benefit”, that is, the pursuit of people’s maximization of health benefits. Cato et al. used the notion of “benefit maximization” to explain the altruism involved in individuals’ preventative health behaviors during the epidemic. These authors pointed out that benefits as a whole are composed of both “benefits” and “costs”; this notion contains both material and psychological benefits and costs, and people choose to engage in preventative health behaviors by balancing the benefits and the costs. Compliance with government-mandated preventative health behaviors reduces not only the possibility of becoming infected oneself but also the probability of infecting others. Preventative health behaviors can benefit others aside from the individuals who engage in the behaviors, and these social benefits are greater than individuals’ personal benefits. As such, although individuals may have to pay a cost to engage in such behaviors (such as changing their daily habits or enduring the loneliness entailed by social isolation), the measured benefits are still higher than the costs. In the pursuit of utility maximization, individuals consistently adopt preventative health behaviors that are both selfish and altruistic [18].

### 1.1. Factors Influencing Altruistic Behaviors

Individual differences in demographic characteristics, especially gender differences, may influence altruistic behavior. Regarding gender stereotypes, women are generally considered compassionate, helpful, and generous, while men are considered competitive and aggressive [19]. Xiao et al. conducted a meta-analysis of 46 documents from sample geographical regions in Europe, the United States, Asia, and other non-Western traditional cultures and found that, although gender differences were generally small, when differences were found, women were more likely to behave altruistically and prosocially in emergency situations [20]. Furthermore, from the concept of risk awareness, women are generally more aware of the risks associated with the disease, so they take COVID-19 more seriously and adopt preventative health behaviors more aggressively than men [6,21,22]. Accordingly, this study proposes the following hypothesis:

**Hypothesis** **1.** *Women exhibit more preventative health behaviors with respect to COVID-19 than men*.

Fear is a common emotion expressed by individuals during disease outbreaks, and studies have pointed out that during infectious disease outbreaks, fear of infection can trigger individuals’ defensive motivations [23,24], which in turn prompts individuals to engage in altruistic behaviors [25]. In particular, when an individual’s fear of infection increases, the psychological costs also increase. The individual’s perceived expected benefits from protecting others are also relatively large, and the individual’s willingness to engage in preventative health behaviors also increases [26].

In fact, studies have shown that anxiety encourages individuals to seek safety-promoting behaviors to protect themselves [27,28,29], and one strategy for self-protection is altruistic behavior. Chan explained from the perspective of biological evolution that our human ancestors obtained evolutionary benefits from helping others who had nothing to do with themselves, which is a manifestation of reciprocal altruism; accordingly, people need to establish connections with others to cope with the current situation of danger [30]. Shah et al. similarly noted that individuals take actions to mitigate and soothe the suffering of others to alleviate their own pain and fear [25]. Individuals can experience the same emotional responses by witnessing the emotions of others; simply seeing someone who is fearful may increase the individual’s own level of fear, thus prompting individuals to act altruistically and unite to face threats or dangers [30]. Accordingly, this study proposes the following hypothesis:

**Hypothesis** **2.** *Individuals with higher anxiety concerning COVID-19 are more inclined to adopt preventative health behaviors*.

### 1.2. Effects of Altruistic Behaviors

Altruistic behaviors have been found to increase individuals’ positive emotions, such as happiness and well-being [14,31]. From a medical perspective, Post noted that although the primary purpose of public health is the mitigation of disease and dysfunction, the primary prevention stage usually involves efforts to improve human well-being through prosocial and altruistic behaviors. It is believed that altruism can provide happiness to individuals, including by inspiring positive emotions such as hope, happiness, and good feelings about themselves, which can replace harmful personal negative emotions and thus achieve the purpose of primary prevention [32]. From the perspective of neuroscience, Lozada, D’Adamo, and Fuentes found that when individuals help others, the areas of the brain that are activated are the same as those that are activated when individuals receive rewards or experience happiness; helping behavior and emotional bonds can produce certain related neuropeptides and hormones that reduce stress and anxiety [33]. Therefore, from an altruistic point of view, preventative health behaviors contribute to the acquisition of mental health benefits in addition to physical health benefits for the individual.

Subjective well-being is a key indicator of an individual’s positive development and mental health [34]. The World Health Organization (WHO) defines mental health as a state of well-being in which everyone is able to achieve their potential, overcome the stresses of everyday life, work productively, and contribute to their communities [35]. According to this definition, well-being includes life satisfaction, positive emotions, and the appropriate functioning of personal activities and social life [36]; as such, well-being is a multidimensional concept. The mental health continuum proposed by Keyes, which combines two views of happiness, a hedonic view and a eudaimonic view, divides subjective well-being into the three types of emotional well-being (EWB), psychological well-being (PWB) and social well-being (SWB) [37]. EWB involves the concept of hedonism, which equates happiness with positive emotions, such as personal pleasure and happiness, as well as life satisfaction. According to the eudaimonic perspective, the sense of well-being involved in happiness is the result of virtuous activities and the pursuit of meaning in life. In this context, well-being indicates good life functions, not simply the experience of positive emotions. Of such functions, those that are related to personal growth and achievement at the individual level are considered elements of PWB, and those that are related to social-level goals and a commitment to shared values are considered aspects of SWB [15,35,38,39].

Xi et al. found that altruistic behavior can explain more than 50% of the variance in subjective well-being [39]. An experimental study by Lu et al. also found that an intervention including related educational measures intended to promote altruistic behavior among adolescent students can help improve students’ life satisfaction and positive emotions [34]. Osumi and Yamane explained the individual’s altruistic behavior toward nonfamily members from the perspective of reciprocal altruism, which helps improve the individual’s subjective well-being [40].

Although few studies have examined the influence of preventative health behaviors with respect to infectious diseases on individual subjective well-being, this study proposes the following hypothesis based on previous research concerning altruistic behavior and subjective well-being:

**Hypothesis** **3.** *Individual preventative health behaviors are positively predictive of well-being*.

Well-being, which is related to positive emotions, is reasonably expected to be negatively related to negative emotions such as depression. Therefore, altruistic behavior is not only expected to increase the individual’s well-being but also to reduce negative emotions such as individual depression [15,31,32]. Depression and anxiety, although they often overlap clinically and exhibit comorbidity, are different [41,42]. Anxiety is a complex emotional response that includes tension, fear, panic, and worry [43]. Anxiety may arise when an individual experiences a high degree of uncertainty regarding whether impending physical or psychological harm can be prevented [44]. Anxiety is often future-oriented, situational, and probabilistic. Depression is a perception of failure and loss. When a sense of helplessness with respect to negative events becomes a sense of hopelessness over time, depression may arise [42], which includes disordered states of unhappiness, meaninglessness, worthlessness, hopelessness, and suicidal thoughts [45].

Although altruistic behavior is reasonably expected to improve individual well-being and thereby reduce negative emotions such as depression, the findings of empirical studies have been inconsistent. Miller et al. claimed that altruism can help prevent the onset of depression in high-risk groups with a family history of depression [46]. However, Fujiwara found that people who displayed altruism were more likely to suffer from major depression. Fujiwara inferred that individuals who have been raised to have high moral standards may be inclined to behave altruistically; however, they may seek to fulfill unrealistic moral codes that are disconnected from real life, which causes them to feel sad or powerless regarding their own lives, thus leading to a higher risk of major depression [47].

Fujiwara’s study further noted that the impact of altruistic behavior on major depressive disorder varies by type of altruistic behavior, and that individuals who provide financial support to nonfamily members may be at risk of major depression, but that unpaid human help and emotional support were not associated with episodes of major depressive disorder [48]. Given the unclear relationship between altruistic behavior and depression, this study raised the following research question:

***Question 1:** How are individual preventative health behaviors related to depression?*

## 2. Methods

### 2.1. Participants

To comply with the government’s COVID-19 prevention regulations, this study adopted an online questionnaire to avoid interpersonal contacts. The survey used the *SurveyCake* online questionnaire, and data were collected from 17 May to 11 June 2020. A total of 340 questionnaires were recovered; among them, there were 136 male and 204 female participants. The age ranged from 15 to 66 years old, and the average age was 26.72 years old (SD 10.5, median 22).

### 2.2. Instruments

#### 2.2.1. Well-Being

The well-being scale was based on the mental health continuum-short form (MHC-SF) [49,50] and included three dimensions (i.e., emotional well-being, psychological well-being, and social well-being). Emotional well-being included three items that covered positive emotion and satisfaction with life. Psychological well-being included six items that covered self-acceptance, personal growth, purpose in life, environmental mastery, autonomy, and positive relations with others. Social well-being included five items that covered social acceptance, social actualization, social contribution, and social integration. Cronbach’s alpha reliability test results of this scale were emotional well-being (α = 0.91), psychological well-being (α = 0.90), and social well-being (α = 0.82).

#### 2.2.2. Depression

The Center of Epidemiology Study-Depression Scale (CES-D) [51] and the current COVID-19 situation were referenced to develop a depression scale more suitable for this study. The researchers adopted principal components analysis (PCA) extraction of exploratory factor analysis (EFA) and used varimax rotations; then, based on the eigenvalues that were higher than 1 to set the extraction standard of the connotation dimension, a factor loading higher than 0.50 was used as the threshold for selecting items. The results of the Kaiser–Meyer–Olkin (KMO) test was 0.91, and Bartltett’s sphericity test reached significance (χ^2^_(55)_ = 1871.36, *p* < 0.001). The scale extracted two factors; the total explainable variance was 62.98%. Factor one, named negative self-perceptions, included 8 items (α = 0.90), and indicated individuals’ negative feeling levels during the COVID-19 pandemic (e.g., I felt sullen during the pandemic.) Factor two was named negative perception of life, and included 3 reverse-scored items (α = 0.78), and indicated the level of negative feelings about life during the pandemic (e.g., I enjoyed life during the pandemic).

#### 2.2.3. COVID-19 Health Preventative Behavior

To understand how the participants responded to the relevant COVID-19 preventative behaviors, the researchers, based on previous studies, compiled the COVID-19 health preventative behavior scale [2,52,53]; moreover, its content and validity were also verified by experts. The scale adopted a five-point Likert scale ranging from “completely disagree” to “completely agree” for 1 to 5 points, respectively. The EFA results yielded a KMO test value of 0.86, and Bartltett’s sphericity test reached significance (χ^2^_(66)_ = 1955.38, *p* < 0.001). The scale extracted three factors, and the total variance explained was 68.35%. Factor one was named epidemic prevention hygiene habits, included 5 items, and indicated the level of emphasis on hygiene and hygiene etiquette (e.g., I would try to avoid coughing when there are people around me.). Factor two was named keeping social distance, included 4 items, and indicated the maintenance social distance and reducing interpersonal contacts (e.g., I would cancel or postpone dining out with my friends.). Factor three was named helping others mitigate the epidemic, included 3 items, and indicated the willingness to donate substantial or non-substantial resources to help others (e.g., If I won’t need to use surgical masks, I am willing to give the masks to those who need them). These items are preventive health behaviors that are not mandated but encouraged by the Taiwanese government. The reliability of Cronbach’s alpha for each aspect was 0.86, 0.83, and 0.83.

#### 2.2.4. COVID-19 Anxiety Perception

This study used two questionnaire items, “I am very worried about getting COVID-19” and “I am very worried about my family and friends getting COVID-19”, which were from the two aspects of “worry oneself will be infected” and “worry one’s relatives and friends will be infected”, to investigate the participants’ levels of COVID-19 anxiety. The options of the item ranged from “completely disagree” to “completely agree” for 1 to 5 points, respectively. In this investigation, the participants’ scores for “worry oneself will be infected” were *Mean* = 3.18 and *SD* = 1.11, while the scores for “worry one’s relatives and friends will be infected” were *Mean* = 3.50 and *SD* = 1.06. The scores of both aspects were higher than the average score (i.e., 3 points), indicating that the participants had a certain level of anxiety about being infected with COVID-19. Dependent sample *t* test analysis showed that the participants’ anxiety level for the “worry one’s relatives and friends will be infected” aspect was significantly higher than that of the “worry oneself will be infected” aspect, where *t*_(339)_ = −8.41, *p* < 0.001, *d* = 0.30, with a small effect size [54].

### 2.3. Data Processing and Analysis

All analyses were performed using SPSS 22.0 software (IBM Corp, Armonk, NY, USA). This paper adopted an independent *t* test to test the differences between gender and COVID-19 preventative health behaviors to test hypothesis 1 and then adopted regression analysis to explore the predictive effects of COVID-19 anxiety perception on preventative health behaviors and COVID-19 preventative health behaviors on well-being and depression to test hypotheses 2 and 3 and answer research question 1.

### 2.4. Ethical Considerations

This study followed the code of research ethics and conformed to the Taiwan government’s institutional review board rules for exempt review. We did not collect any relevant identifying information from the participants, and an anonymous design questionnaire was used in this study. Four checkpoints are provided in the research design so that respondents can stop answering at any time. (1) The instructions in the invitation email link to the online questionnaire clearly informed the participants of the research purpose and their rights regarding joining or dropping out of this study at any time during online participation. (2) The respondents decided to participate in the study after reading about the study purpose. (3) During online participation, the respondents are allowed to quit at any time if they wish to, without fear of penalty; (4) Upon completing the entire questionnaire and pressing “Send”, the respondents were assured that their participation was voluntary, anonymous, and strictly confidential and that they had the right to refuse to participate in the study at any time without any penalty.

## 3. Results

### 3.1. Gender Differences in COVID-19 Preventative Health Behaviors

Table 1 indicates that gender had a significant impact on aspects of preventative health behaviors; among them, the female participants’ performances on epidemic prevention hygiene habits, maintaining social distance, and helping others mitigate the epidemic were significantly higher than those of the male participants. The results support Hypothesis 1.

### 3.2. The Prediction of COVID-19 Anxiety Perception to Preventative Health Behaviors

Table 2 shows that both “epidemic prevention hygiene habits” (*F* = 11.55, *p* < 0.001) and “maintaining social distance” (*F* = 5.43, *p* = 0.0047) have a significant positive linear relationship with the regression model of the two independent variables of COVID-19 anxiety perception (i.e., “worry oneself will be infected” and “worry one’s relatives and friends will be infected”). However, each independent variable is not statistically significant, indicating that the independent variables of “worry oneself will be infected” and “worry one’s relatives and friends will be infected” have no significant influences on “epidemic prevention hygiene habits” or “keeping social distance”. Moreover, the regression model of “helping others mitigate the epidemic” and COVID-19 anxiety perception was not statistically significant (*F* = 0.89, *p* = 0.41). The results partially support Hypothesis 2.

### 3.3. The Prediction of COVID-19 Preventative Health Behaviors to Well-Being

Table 3 shows that “epidemic prevention hygiene habits” is significantly related to “psychological well-being”, where *β* = 2.42, *p* = 0.02, which indicates that better performance of epidemic prevention hygiene habits will increase emotional well-being. “Keeping social distance” is significantly associated with “emotional well-being” (*β* = −3.19, *p* = 0.002) and “social well-being” (*β* = −2.63, *p* = 0.009), which means that when the participants perform better in “keeping social distance”, their sense of “emotional well-being” and “social well-being” will be lower. “Helping others mitigate the epidemic” has significant positive correlations with “psychological well-being” (*β* = 4.20, *p* < 0.001) and “social well-being” (*β* = 3.09, *p* = 0.002), which implies that the better performance of “helping others mitigate the epidemic” will induce a higher sense of “psychological well-being” and “social well-being”. The positive predictive relationship between “epidemic prevention hygiene habits”, “helping others mitigate the epidemic”, and well-being supports Hypothesis 3; however, the findings of “keeping social distance” and well-being are opposite to Hypothesis 3.

### 3.4. The Prediction of COVID-19 Preventative Health Behaviors against Depression

Table 4 shows that “helping others mitigate the epidemic” has significant correlations with “negative self-perceptions” and “negative perception of life”; in other words, with better performance in “helping others mitigate the epidemic”, the negative perceptions in “negative self-perceptions” (*β* = −2.46, *p* = 0.02) and “negative perception of life” (*β* = −2.24, *p* < 0.001) will be lower.

## 4. Discussion

Many scholars claim that preventative health behaviors by the public, such as wearing masks, maintaining social distance, and hand hygiene, are practical and low-cost means of mitigating the spread of disease during the COVID-19 epidemic. The focus of these protective measures is altruism, which actively involves every citizen and serves as a symbol of social solidarity in the global response to the epidemic [4]. Therefore, this study explained the preventative health behaviors employed by individuals to prevent COVID-19 infection from an altruistic perspective and explored the characteristics and effects of various manifestations of altruism in preventative health behaviors with respect to COVID-19.

### 4.1. Gender and Preventative Health Behaviors for COVID-19

The results of this study show that women are more inclined to adopt preventative health behaviors than men. Previous studies have noted that women are generally more aware of the risks of disease, which may be due to the nature of human biological evolution. Since many women spend a great deal of their lives caring for children, face substantial risks from disease and death during pregnancy and childbirth, and pay a high price to reproduce, they face greater evolutionary pressure in this context than men [6]. To reduce the psychological cost of stress, women are more inclined to avoid the risk of pathogen infection. Furthermore, women are considered compassionate, caring, cooperative, helpful, and prosocial [19,20] and may therefore be more inclined to adopt preventative health behaviors to protect themselves and others. However, from the perspective of gender stereotypes, these traits may be derived from social norms and expectations rather than gender differences caused by nature, and the pressure entailed by these social norms and expectations imposes psychological costs on women [55]. Heilman and Chen found that refusing to exhibit altruistic behavior reduced evaluative favorability in women but not men, noting that gender stereotype prescriptions about how men and women should behave can lead to different evaluative responses to the same altruistic behavior [56]. Brañas-Garza et al. also found that men expect women to be more altruistic than men and that women self-identified that they should be more altruistic than men [57]. Follow-up research could investigate whether perceptions of social norms and expectations have an impact on gender adherence to preventative health behaviors.

### 4.2. Anxiety Perception and Preventative Health Behaviors

Regarding the relationship between anxiety and preventative health behaviors, the study shows that the more anxiety individuals experience with respect to contracting COVID-19, the more likely they are to adopt preventative health habits and maintain social distance. No significant relationship was found between preventative health behaviors related to helping others mitigate the epidemic and anxiety. From a utilitarian perspective, benefit is a measure that takes into account the individual’s material and psychological benefits and costs [17,18]. An individual’s fear of disease infection may have a psychological cost, which in turn prompts the individual to engage in corresponding preventive health behaviors [25,26]. However, the measures required by these preventative health behaviors may also increase the psychological and material costs individuals face, such as inconvenience resulting from changes in daily living habits [18] or the loss of personal resources from donating materials or providing unpaid services, which reduces individuals’ probability of engaging in preventative health behaviors. Furthermore, altruism is not an all-or-nothing phenomenon but exhibits varying degrees with respect to individual benefits and costs [58]. It may include the pure altruism of genuine selflessness, weak altruism (altruism that does not include any positive intention to benefit others) or contingent/conditional altruism that depends on personal gains. Ref. [59], all of which may be affected by the individual’s perceptions of various benefits and costs.

In this study, the preventative health behaviors associated with donating medical masks and assisting others with epidemic mitigation needs may be costlier to individuals than the preventative health behaviors involved in adopting hygiene habits to mitigate the epidemic and maintaining social distancing. For example, donating medical masks may lead to a shortage of medical masks. Therefore, when measuring the benefits and disadvantages of anxiety with respect to different preventative health behaviors, individuals with high anxiety regarding the possibility of being infected with COVID-19 may feel that the benefits of hygiene habits intended to mitigate the epidemic and those of maintaining social distancing outweigh the costs, leading to better preventative health performance. For individuals who are motivated purely by altruism, the costs of helping others mitigate the epidemic may not always ensure the benefits of alleviating their anxiety with respect to contracting COVID-19, so the predicted relationship between the two factors is not obvious.

### 4.3. Preventative Health Behaviors and Well-Being

According to the results of this study, the better participants’ behavioral performance with respect to anti-epidemic hygiene habits is, the higher their levels of psychological well-being, and the more behaviors they exhibit to help others mitigate the epidemic, the higher their levels of psychological and social well-being. Psychological well-being involves a belief in self-development, such that individuals believe that happiness is based on personal growth and becoming a better person. In contrast, social well-being involves making contributions to others; that is, individuals focused on social well-being believe that happiness is based on helping others and creating a better society [15,34,38]. It is reasonable to expect that altruistic behavior is positively associated with psychological well-being and social well-being [15] because altruistic behavior includes acts intended to help others, thereby allowing individuals to become better people who believe that benefitting the world is a reward in itself [59]. Such behavior thus provides intrinsic psychological benefits to the individual.

Notably, participants in this study performed better with respect to preventative health behaviors that required them to maintain social distancing, resulting in lower levels of social well-being and emotional well-being. As noted by Heffner, extreme social distancing is the most damaging preventative health behavior [5]. Social relationships are an important resource for human survival; humans rely on each other to obtain what they want and need, and this sense of social interdependence produces a strong desire for social connection, which is extremely important to human well-being [60]. In particular, this sense reflects the social well-being produced by an individual’s contribution to society. Social well-being focuses on the social nature of life and recognizes that people are inherently social animals and that social relationships are a source of human happiness. Therefore, social integration, interaction and relationships with others belong to the category of social well-being [61]. Accordingly, maintaining social distancing stands in opposition to these social connections and is not conducive to the individual’s social well-being.

Furthermore, emotional well-being involves a belief in enjoyment that equates well-being with personal happiness and functions as a personal, emotional perception that one will experience happiness and avoid pain. When people base their well-being primarily on personal happiness, they are less likely to pursue happiness through altruistic behavior, which may reduce their positive emotions (e.g., from providing benefits to others) and increase negative emotions (e.g., by inflicting personal losses) [15]. However, the preventative health behaviors involved in social distancing require the individual to sacrifice certain pleasant activities, such as going out for parties or dinners [18], which may decrease individuals’ emotional well-being.

### 4.4. Preventative Health Behaviors and Depression

Regarding the relationship between preventative health behaviors and depression, this study showed that participants engaged in behaviors intended to help others mitigate the epidemic, which helped reduce depression, while epidemic prevention hygiene habits and social distancing had no significant correlations with depression, which is in line with the view expressed by Fujiwara that different altruistic behaviors have different effects on depression. Furthermore, the research results show that helping others mitigate the epidemic can help reduce depression [48], a similar conclusion to that of Miller et al., who claimed that altruistic behavior helps individuals suppress depression.

As mentioned above, regarding the relationship between preventative health behaviors and well-being, behaviors intended to help others mitigate the epidemic may cause individuals to feel that they are better people and that they can bring beauty to the world [14], thereby reducing depression, negative self-perceptions and negative perceptions of life. This situation is also in line with the fact that altruistic behavior not only helps increase individual happiness but also reduces negative emotions such as depression in individuals [15,31,32].

However, the results of this study are different from the findings reported by Fujiwara, who claimed that altruists are more likely to suffer from major depression. A possible reason for this is that the altruistic situations investigated by Fujiwara’s study tended to be purely altruistic behaviors, such as paying more for health insurance so that everyone can acquire health care, volunteering time or money to promote social welfare, or raising one’s own taxes to help others. Such unidirectional, prosocial, and altruistic behavior may lead to demands for individuals to lead a highly moral life, thereby increasing the risk of depression [47]. The preventative health behaviors explored in this study tended to be reciprocal altruistic behaviors; even though individuals must pay a cost to engage in such behaviors, they can still benefit from them as well [16,18]. Therefore, while helping others prevent the spread of the virus may require personal costs and sacrifices, such assistance is not only able to reduce the chances of others becoming infected but also able to prevent community infection and reduce one’s own risk of exposure to COVID-19. This benefit, in turn, helps reduce an individual’s perception of depression during the epidemic.

Finally, it is important to note the small size of the effect found for the significant associations among preventative health behaviors and anxiety, well-being and depression, which may be due to the fact that the impact of the COVID-19 outbreak on Taiwan was not as serious as its effects in other countries. As of May 2021, the epidemic situation in Taiwan was severe, and the alert level was raised to the third level, requiring the implementation of epidemic mitigation and restriction measures, such as wearing a mask when going out; the closure of leisure and entertainment venues; the cessation of operations by restaurants and film companies; and restrictions on weddings, funerals, celebrations, and religious activities. Therefore, it may be the case that the temporal and spatial backdrop against which the survey was administered reduced the impact of the COVID-19 epidemic on individuals’ perceptions of happiness, depression or anxiety at that time, thereby leading to the variables referenced by this study exhibiting only a small effect size. Park et al. also noted that people’s optimistic biases with respect to the epidemic can reduce their anxiety, fear and risk perceptions regarding the epidemic, which in turn affects their willingness to engage in personal preventative health behaviors [62]. Therefore, the question of whether individuals’ perceptions of the severity of the epidemic affect the magnitude of the effect demonstrated between the variables referenced by this study remains to be explored by subsequent studies.

However, when advocating public adherence to preventative health behaviors, it is also important to note that different preventative measures may have different effects on people’s emotions, especially with respect to preventative health behaviors related to social distancing. Social distancing stands in opposition to the inherently social nature of human beings, causing people to feel emotionally unhappy, isolated, etc. This practice can be described as the most destructive preventative health behavior. However, during the outbreak of infectious diseases, social distancing and other preventative behaviors are regarded as important and effective measures with respect to preventing infection. Therefore, when implementing social distancing-related restrictions during the epidemic, it is also necessary to consider how to alleviate the negative emotions of restricted actors and to pay attention to their degree of life satisfaction.

## 5. Limitations

Regarding the limitations of this study and possible future directions for research, first, the preventative health behaviors explored in this study are examined via the results of participants’ self-reports rather than objective observations; thus, they may be easily affected by participants’ personal subjective biases, and the behaviors reported by participants may not be consistent with their actual behaviors. Moreover, our means of assessing preventative health behaviors intended to help others mitigate the epidemic tend to ask participants about their behavioral intentions rather than their actual behaviors. Although many studies have proven that behavioral intentions and behaviors are highly correlated, there may still be differences between the two. Therefore, it is suggested that future research can switch to observational studies or ask participants about their actual actions and their experiences with different preventative health behaviors to obtain a more precise understanding of participants’ actual preventative health behaviors. In addition, although preventative health behaviors can be considered manifestations of altruistic behaviors, the questions of whether these ostensibly altruistic behaviors are actually motivated by altruism or whether participants perceive the altruistic significance of preventative health behaviors are worth further exploration. Therefore, it is recommended that subsequent research also explore related variables such as altruistic motivation, identity and awareness. Exploration of these variables may also contribute to identifying whether preventive health behaviors are driven by anxiety perception or altruism-related psychological factors. Despite these research limitations, we believe that exploring the relationships among preventative health behaviors, demographic characteristics and aspects of mental health, such as well-being, depression, and anxiety, from an altruistic perspective in the context of the COVID-19 epidemic is an important contribution and can provide us with insights into the complex relationships among preventative health behaviors, demographic characteristics, and mental health.

## 6. Conclusions

The results of this study indicated that females had better preventive health behaviors, including hygiene habits, social distancing and helping others prevent the epidemic, than males. Anxiety about COVID-19 infection led individuals to adopt hygienic habits and social distancing instead of helping others prevent the epidemic. Hygiene habits and helping others prevent epidemics improved the individual’s psychological well-being or social well-being, and helping others control the epidemic contributed to furthermore reducing individual depression. However, the preventive health behavior of social distancing was not conducive to emotional well-being and social well-being. Given that affective elements are related to individual behaviors, the use of prosocial altruistic language may play an important role in advocating people to comply with COVID-19 preventive health behaviors. In addition, it is worth noting that different preventive health behaviors may have different effects on people’s mental health, especially when implementing social distancing-related epidemic prevention behaviors. It must be carefully considered how the negative psychology of restricted actors can be avoided and how to pay attention to their satisfaction with life.

## Figures and Tables

**Table 1:** *t* test results of the effect of gender on COVID-19 preventative health behaviors. (df = 338).

	*M* (*SD*)		*t*	*p*	*d*
	Male	Female			
Epidemic prevention hygiene habits	4.10 (0.65)	4.43 (0.54)	−5.08 **	<0.001	0.56
Keeping social distance	3.48 (0.89)	3.76 (0.84)	−3.00 **	0.003	0.33
Helping others mitigate the epidemic	3.82 (0.76)	3.99 (0.79)	−1.97 *	0.049	0.22

Figures in bold indicate significance, * *p* < 0.05, ** *p* < 0.01.

**Table 2:** Regression results of COVID-19 anxiety perception to preventative health behaviors.

	Preventative Health Behaviors	Epidemic Prevention Hygiene Habits			Keeping Social Distance			Helping Others Mitigate the Epidemic		
COVID-19 Anxiety		*β*	*t*	*p*	*β*	*t*	*p*	*β*	*t*	*p*
Worry oneself will be infected		0.12	1.32	0.19	**0.15**	1.71 *	**0.09**	−0.09	−0.99	0.33
Worry one’s relatives and friends will be infected		**0.15**	1.73 *	**0.09**	0.03	0.33	0.74	0.12	1.33	0.19
		*R*^2^ = 0.06, Adj *R*^2^ = 0.06			*R*^2^ = 0.03, Adj *R*^2^ = 0.03			*R*^2^ = 0.01, Adj *R*^2^ = 0.01		
		*F* = 11.55 ***, *p* < 0.001			*F* = 5.43 **, *p* = 0.0047			*F* = 0.89, *p* = 0.41		

Figures in bold indicate significance, * *p* < 0.05, ** *p* < 0.01, *** *p* < 0.001.

**Table 3:** Regression results of COVID-19 preventative health behaviors to well-being.

	Well-Being Preventative	Emotional Well-Being			Psychological Well-Being			Social Well-Being		
Health Behaviors		*β*	*t*	*p*	*β*	*t*	*p*	*β*	*t*	*p*
Epidemic prevention hygiene habits		0.004	0.06	0.95	**0.15**	2.42 *	**0.02**	0.04	0.61	0.54
Keeping social distance		**−0.21**	−3.19 **	**0.002**	0.01	0.10	0.92	**−0.17**	−2.63 **	**0.009**
Helping others mitigate the epidemic		0.01	0.08	0.93	**0.25**	4.20 ***	**<0.001**	**0.19**	3.09 **	**0.002**
		*R*^2^ = 0.04, Adj *R*^2^ = 0.03			*R*^2^ = 0.12, Adj *R*^2^ = 0.11			*R*^2^ = 0.04, Adj *R*^2^ = 0.03		
		*F* = 4.80 **, *p* = 0.003			*F* = 14.81 ***. *p* < 0.001			*F* = 4.52 **, *p* = 0.004		

Figures in bold indicate significance, * *p* < 0.05, ** *p* < 0.01, *** *p* < 0.0001.

**Table 4:** Linear regression of COVID-19 preventative health behaviors against depression.

	Depression	Negative Self-Perceptions			Negative Perception of Life		
Preventative Health Behaviors		*β*	*t*	*p*	*β*	*t*	*p*
Epidemic prevention hygiene habits		−0.09	−1.34	0.18	−0.03	−0.43	0.67
Keeping social distance		0.01	0.12	0.91	0.03	0.46	0.65
Helping others mitigate the epidemic		**−0.15**	−2.46 *	**0.02**	**−0.24**	−3.97 ***	**<0.001**
		*R*^2^ = 0.04, Adj *R*^2^ = 0.03			*R*^2^ = 0.06, Adj *R*^2^ = 0.05		
		*F* = 4.61 **, *p* = 004			*F* = 6.90 ***, *p* < 0.001		

Figures in bold indicate significance, * *p* < 0.05, ** *p* < 0.01, *** *p* < 0.001.

## Data Availability

The dataset cannot be reused or provided since our participants agreed to their data being used only for this study.
